# Genetic Diversity of Dengue Virus in Clinical Specimens from Bangkok, Thailand, during 2018–2020: Co-Circulation of All Four Serotypes with Multiple Genotypes and/or Clades

**DOI:** 10.3390/tropicalmed6030162

**Published:** 2021-09-04

**Authors:** Kanaporn Poltep, Juthamas Phadungsombat, Emi E. Nakayama, Nathamon Kosoltanapiwat, Borimas Hanboonkunupakarn, Witthawat Wiriyarat, Tatsuo Shioda, Pornsawan Leaungwutiwong

**Affiliations:** 1 Department of Microbiology and Immunology, Faculty of Tropical Medicine, Mahidol University, Bangkok 10400, Thailand; kanaporn.pol@gmail.com (K.P.); nathamon.kos@mahidol.ac.th (N.K.); 2 Mahidol-Osaka Center for Infectious Diseases (MOCID), Faculty of Tropical Medicine, Mahidol University, Bangkok 10400, Thailand; juthamas@biken.osaka-u.ac.jp (J.P.); emien@biken.osaka-u.ac.jp (E.E.N.); 3 The Monitoring and Surveillance Center for Zoonotic Diseases in Wildlife and Exotic Animals, Faculty of Veterinary Science, Mahidol University, Nakhon Pathom 73170, Thailand; witthawat.wir@mahidol.edu; 4 Department of Viral Infections, Research Institute for Microbial Diseases (RIMD), Osaka University, Osaka 565-0871, Japan; 5 Department of Clinical Tropical Medicine, Faculty of Tropical Medicine, Mahidol University, Bangkok 10400, Thailand; borimas.han@mahidol.ac.th

**Keywords:** Bangkok, clades, co-circulation, DENVs, genetic diversity, genotypes

## Abstract

Dengue is an arboviral disease highly endemic in Bangkok, Thailand. To characterize the current genetic diversity of dengue virus (DENV), we recruited patients with suspected DENV infection at the Hospital for Tropical Diseases, Bangkok, during 2018–2020. We determined complete nucleotide sequences of the DENV envelope region for 111 of 276 participant serum samples. All four DENV serotypes were detected, with the highest proportion being DENV-1. Although all DENV-1 sequences were genotype I, our DENV-1 sequences were divided into four distinct clades with different distributions in Asian countries. Two genotypes of DENV-2 were identified, Asian I and Cosmopolitan, which were further divided into two and three distinct clades, respectively. In DENV-3, in addition to the previously dominant genotype III, a cluster of 6 genotype I viruses only rarely reported in Thailand was also observed. All of the DENV-4 viruses belonged to genotype I, but they were separated into three distinct clades. These results indicated that all four serotypes of DENV with multiple genotypes and/or clades co-circulate in Bangkok. Continuous investigation of DENV is warranted to further determine the relationship between DENV within Thailand and neighboring countries in Southeast Asia and Asia.

## 1. Introduction

Dengue is an arthropod-borne viral disease that occurs and spreads in urban and rural areas of tropical and subtropical regions and greatly affects human health and economics [1,2,3]. Dengue is transmitted via the mosquito species *Aedes aegypti* and *A. albopictus* [4,5]. More than 75% of the global population is at risk of dengue infection, and this risk has increased considerably due to increases in travel and globalization [6,7]. In most cases, dengue infection is asymptomatic or causes an acute febrile illness or dengue fever, although a small proportion of patients may develop more severe clinical manifestations, such as dengue hemorrhagic fever (DHF) or dengue shock syndrome (DSS) [8,9,10]. The Bureau of Epidemiology in the Ministry of Public Health (MoPH) of Thailand reported more than 100 infections per 100,000 population, with 111, 133, and 51 deaths in 2018, 2019, and 2020, respectively [11,12]. Currently the dengue incidence in Thailand is high throughout the country [13], including the Bangkok metropolitan area. Bangkok, which is the capital city of Thailand, is located in the center of the country and serves as a transportation hub. Dengue fever cases have been reported in Thailand and Bangkok since the 1950s [14,15] and exhibit a spatial-temporal circulation pattern throughout the country [16].

Dengue is caused by dengue viruses (DENVs) belonging to the genus *Flavivirus*, family *Flaviviridae* [6]. DENV is an enveloped virus containing a single-stranded RNA with positive polarity of approximately 10.6 kb in length as the genome. DENV genomic RNA encodes three structural proteins (capsid (C), premembrane/membrane (prM), and envelope (E) proteins) and seven nonstructural (NS) proteins (NS1, NS2A, NS2B, NS3, NS4A, NS4B, and NS5) [17]. Based on antigenic differences, DENVs are divided into four serotypes, DENV-1, DENV-2, DENV-3, and DENV-4, which elicit only limited cross-protection immunity [18,19]. Each serotype is further divided into several genotypes according to geographical distribution and epidemic potential [20,21]. Currently, DENV-1 consists of five (I–V) genotypes [21,22]. Genotype I originated in Asia, whereas genotype II represents Thailand strains. Genotype III viruses are sylvatic strains. Genotype IV viruses are from the South Pacific region, whereas genotype V viruses are from the Americas and Africa. DENV-2 consists of six genotypes [23]. The Asian/American genotype previously dominated in Southeast Asia and now circulates in Central and South America. The Asian I and Asian II genotypes circulate in Asia. The Cosmopolitan genotype exhibits a wide-spread distribution around the world [24]. The American genotype is predominant in and specific to Central and South America. The sylvatic genotype is frequently found in non-human primates from African and southeast Asian forests. DENV-2 is known to be associated with severe dengue cases [24,25]. DENV-3 consists of five genotypes [26]. Genotype I is present in Southeast Asia and the South Pacific, and genotype II originated in Thailand. Genotype III was originally isolated in the Indian subcontinent, and genotype IV was originally isolated in the Americas. Genotype V is found in both Asia and the Americas. DENV-4 also consists of five genotypes [23]. Genotype I was first reported in the Philippines in 1956 (prototype), and genotype IIA is distributed in Southeast Asia and China. Genotype IIB was isolated in Southeast Asia and the Pacific islands, and genotype III has only been reported in Thailand since 1997. Sylvatic DENV-4 is found exclusively in non-human primates [27].

Recently, the MoPH reported serotyping results for 88 DENV-associated deaths in Thailand in 2019. DENV-2 was found in 51% of the deaths followed by DENV-1 (35%), whereas the percentages of DENV-4 and DENV-3 were low [12]. In 2020, DENV-2 continued to predominate in terms of the percentage of deaths (65.7%), followed by DENV-1 and DENV-4, but there were no deaths associated with DENV-3 [11,12]. In Bangkok, during the period between 1973 and 2012, incidence data indicated that DENV-4 was dominant only in 1993–1994, whereas DENV-1, -2, and -3 co-circulated and predominated in turns [28].

Phylogenetic analyses of the envelope region of DENVs have been used to determine the viral genotypes and evolutionary relationship between past and present isolates [21]. The increasing diversity of viral geographic distribution may affect the severity of disease and is therefore an important monitoring parameter for DENV surveillance. Thus, continuous monitoring and surveillance are important for determining the characteristics of current DENV strains and assessing the endemic situation in Bangkok, Thailand, as well as countries bordering Thailand. The data from such programs can be further used to design preventive and control measures targeting DENV circulating in Thailand and Southeast Asia. In the present study, therefore, we examined the genetic diversity of DENV sequences obtained from clinical specimens in Bangkok during the period 2018–2020. We report here the co-circulation of all four serotypes of DENV during this period, with multiple genotypes or clades circulating in Bangkok.

## 2. Materials and Methods

### 2.1. Clinical Sample Collection

Patients who presented to the Hospital for Tropical Diseases with symptoms of fever during 2018–2020 were suspected to have DENV infection and investigated by one of the following three tests: (i) NS1 antigen detection (BIOSYNEX, Illkirch-Graffenstaden, France), (ii) anti-DENV IgM or IgG antibody detection (SD Bioline, Seoul, Korea), or (iii) Dengue Real-time PCR kit (Sacace Biotechnologies, Como, Italy). Patients with a positive result in at least one of the three tests were requested to participate in this study. Clinical blood samples were centrifuged for 10 min at 2000× *g* for serum separation, and serum samples were stored at −80 °C until further analysis. This study was reviewed and approved by the Ethics Committee of the Faculty of Tropical Medicine, Mahidol University, Bangkok, Thailand (TMEC 19-051). This study was exempt from obtaining participants’ consent since only leftover specimens were used after anonymization.

### 2.2. DENV Serotype Determination

Viral RNA was extracted from patient serum samples using a QIAamp viral RNA mini kit (Qiagen, Hilden, Germany) according to the manufacturer’s instructions. DENV infection was confirmed by reverse transcription quantitative/real-time PCR (RT-qPCR) using previously described primer pairs and protocols [29]. DENV-positive samples were then analyzed using a commercial dengue subtyping multiplex kit (Genesig, Chandler’s Ford, UK) for DENV serotype determination according to the manufacturer’s instructions.

### 2.3. Whole Genome Sequencing

cDNA was synthesized from extracted viral RNA using the SuperScript III First-Strand Synthesis System (Invitrogen, Carlsbad, CA, USA) and amplified using Prime Script GXL DNA polymerase (TaKaRa Co., Ltd., Shiga, Japan) and the primers shown in Table S1. The amplified dsDNA was visualized by gel electrophoresis and purified using a Qiagen QIAquick^®^ PCR Purification kit. The concentration of dsDNA was determined using a Qubit dsDNA Assay kit and equalized to 0.2 ng/μL. DNA libraries were prepared using an Illumina Nextera XT library preparation kit (Illumina, San Diego, CA, USA). The library DNA concentration was measured using Bioanalyzer^®^ High-Sensitivity DNA chips (Agilent Technologies, Santa Clara, CA, USA) and then normalized to 4 nM using elution buffer. Sequencing was performed on a MiSeq platform (Illumina) using a MiSeq v2 kit (500 cycles) reagent cartridge. Raw sequence data were generated as FASTQ files. The quality of each sequence was controlled by the default settings for minimum base call quality (Q30), detection threshold (0.1 or 10%), and analysis threshold (0.25 or 25%). The assembled sequences were trimmed and aligned to the references sequences of DENV-1 Mochizuki (AB074760) and DENV-2 16681 (NC 001474). All sequences were submitted to GenBank under accession numbers MZ619036–MZ619041 for DENV-1 and MZ636801–MZ636805 for DENV-2.

### 2.4. Sanger Sequencing

The DENV envelope region was amplified using a One-Step Reverse Transcriptase-PCR kit (Qiagen) with previously designed primers [30,31,32,33,34,35,36,37,38], as shown in Table S2. The products were then subjected to nested PCR (PrimeSTAR GXL DNA polymerase, TaKaRa Co., Ltd.). The PCR products were purified using a QIAquick gel extraction kit (Qiagen). The purified amplicons were sequenced by MACROGEN Company (Seoul, Korea) according to the previously designed serotype-specific primers listed in Table S2. The resulting sequences were analyzed in BioEdit version 7.0.5.3 (Ibis Biosciences, Carlsbad, CA, USA) with the corresponding reference sequences for DENV-1 Mochizuki (AB074760), DENV-2 16681 (NC001474), DENV-3 H87 (M93130), and DENV-4 H241 (AY947539). The assembled sequences of the complete envelope regions determined in the present study were deposited in GenBank under accession numbers MZ618966–MZ619005 for DENV-1, MZ636761–MZ636799 for DENV-2, MZ636813–MZ636820 for DENV-3, and MZ636822–MZ636834 for DENV-4.

### 2.5. Phylogenetic Analysis of DENV Envelope Regions

The 1485 nucleotides covering the complete envelope regions of DENV-1, -2, and -4 and the 1479 nucleotides covering that of DENV-3 were aligned with the genotype reference sequences corresponding to each serotype [30] and the sequences hit using the NCBI Basic Local Alignment Search Tool (BLAST) (http://blast.ncbi.nlm.nih.gov/Blast.cgi, accessed on 13 August 2021) using MUSCLEe in AliView v1.26 [39]. The sequences of the envelope region from GenBank used in the phylogenetic analysis and the DENV sequences from Thailand during the period 2015–2020 are shown in Table S3. The best substitution model was selected using IQ-TREE [40,41,42,43]. A maximum-likelihood (ML) phylogenetic tree was constructed using IQ-TREE and visualized in FigTree v1.4.4 [44].

## 3. Results

### 3.1. Dengue Infection and Serotype Distribution

Of the 1100 suspected DENV cases at the Hospital for Tropical Diseases, Bangkok, Thailand during the period of 2018–2020, 276 patients were identified as DENV positive and agreed to participate in the present study (Table 1). DENV-1 was the predominant serotype throughout the study period at 43.8% overall (121 out of 276 DENV-positive cases), followed by DENV-2, -4, and -3. The circulation of DENV-3 was rare and not detected in 2019 (Table 1). Among serum samples with a low RT-qPCR DENV ct value (<20), six DENV-1 samples (DV1I-TM19-09, -12, -33, -40, -70, and -74) and five DENV-2 samples (DV2A-TM19-13, DV2C-TM19-26, -37, -41, and -80) were selected for whole-genome sequencing using next-generation sequencing (NGS), and the remainder were subjected to Sanger sequencing of the envelope region. In total, 111 E-region sequences were successfully obtained from 46, 44, 8, and 13 samples for DENV-1, DENV-2, DENV-3, and DENV-4, respectively. The ID, GenBank accession numbers, and other details regarding the obtained sequences are show in Table S4.

### 3.2. Phylogenetic Analysis of DENV-1

**Figure 1:**
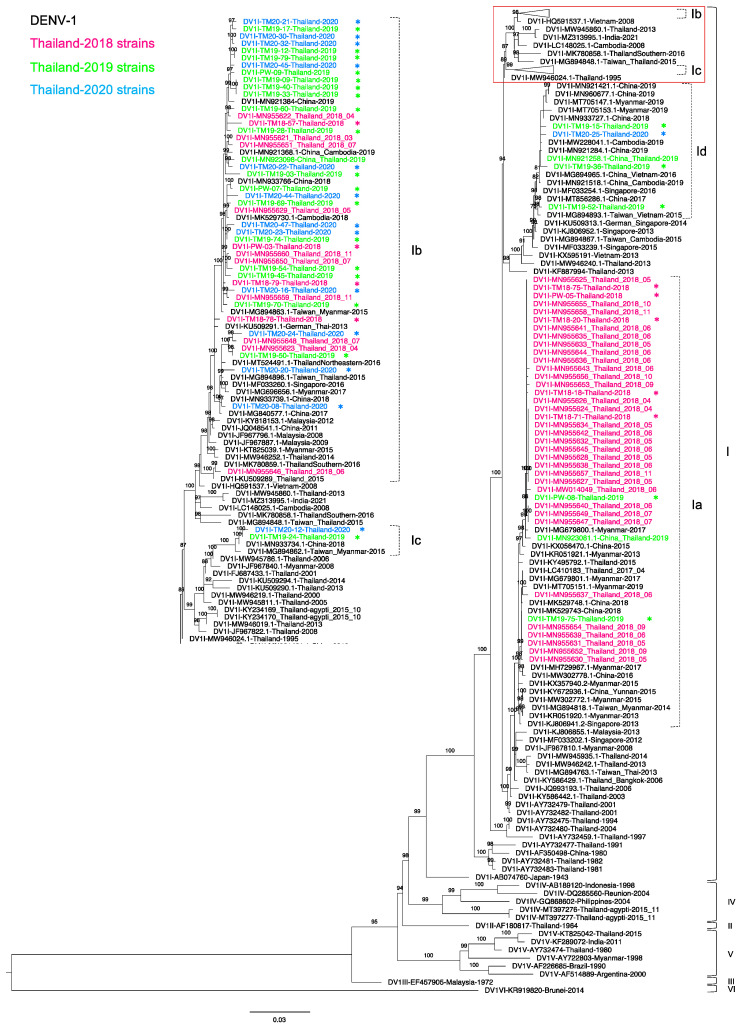
Phylogenetic analysis of Thailand DENV-1 in 2015–2020. The maximum-likelihood (ML) tree was constructed based on the complete envelope (E) region sequences (1485 nucleotides) under TIM2 + F + I + G4 with 1000 ultrafast bootstrap replicates. The ML tree included 46 newly determined sequences of DENV-1 from clinical specimens from Bangkok in 2018–2020, their related strains, Thailand strains during 2015–2020, and the reference genotypes. The sequences collected in 2018, 2019, and 2020 are labeled in pink, green, and blue, respectively, and indicated in the upper left corner. The sequences newly determined in the present study are marked with asterisks (*). Viral genotypes and clades are indicated by black and dotted brackets, respectively, on the right. Sequences are shown by serotype, accession number, country, and reported year. Numbers on branches are bootstrap support values exceeding 80%. Details of the collapsed portion of the tree in the red square in the upper right including clades b and c are shown in the middle.

Among 121 DENV-1-positive samples, we amplified the envelope region of 46 samples comprised of 9, 23, and 14 sequences from 2018, 2019, and 2020, respectively. These newly obtained sequences were compared with previously described recently circulating sequences [30]. In the present study, all DENV-1 sequences belonged to genotype I (DENV-1-I) and were derived from Thailand and Southeast Asia (Figure 1).

The phylogenetic tree revealed that DENV-1-I formed four separate clades. Almost all of the 2018 sequences were in clade a (DENV-1-Ia), which corresponded to lineage A of DENV-1-I in our previous study [30]. This clade included five 2018 strains from our present study. In addition, two of our 2019 strains were also in this clade. Other DENV-1-Ia viruses were derived from Thailand in 2015–2017 [30,32], a Chinese traveler in Thailand in 2019 (MN923081), China in 2015, and Myanmar in 2013–2019 [45]. The phylogenetic tree showed that DENV-1-Ia strains descended from Thailand strains from the 2000s (AY732482) [46,47]. On the other hand, clade b (DENV-1-Ib) formed a vast clade corresponding to lineage B of DENV-1-I in our previous study [30]. This clade contained our DENV-1-I from 2018–2020. Closely related strains included other Thailand strains from 2018 [30], China and Cambodia strains from 2018–2019, and a Myanmar strain from 2015. Moreover, three sequences, DV1-TM19-50, DV1-TM20-20, and DV1-TM20-24, clustered with a sequence from the northeastern part of Thailand from 2016 (MT524491.1). These sequences shared the ancestral strain from Malaysia, Singapore, and Thailand that circulated in 2008–2015. Interestingly, our four sequences from 2019 and 2020 (DV1I-TM19-15, -19-36, -19-52, and -20-25) formed another clade (clade Id) with sequences of viruses isolated from Chinese acute patients in 2017–2019 (MN933727 and MN921421), a Taiwanese who traveled to Cambodia in 2015 (MG894867.1) [48], and from Vietnam in 2015 [49]. The ancestor of this clade was related to the Thailand 2013 strain (KF887994). Furthermore, the remaining two sequences of our 2019 and 2020 viruses (TM19-24 and TM20-12) were in the fourth clade (clade c) with sequences from a Chinese acute patient in 2018 (MN933734.1) and a Taiwanese who traveled to Myanmar in 2015 (MG894862.1) [49]. The ancestor of this cluster was related to the sequences from Thailand in 2006 (MW945786) and Myanmar in 2008 [48]. The phylogenetic tree revealed that the distribution of clade c viruses began in Thailand in 2006 and Myanmar in 2008 and then expanded throughout Southeast Asia [50,51].

### 3.3. Phylogenetic Analysis of Thailand DENV-2 in 2015–2020

**Figure 2:**
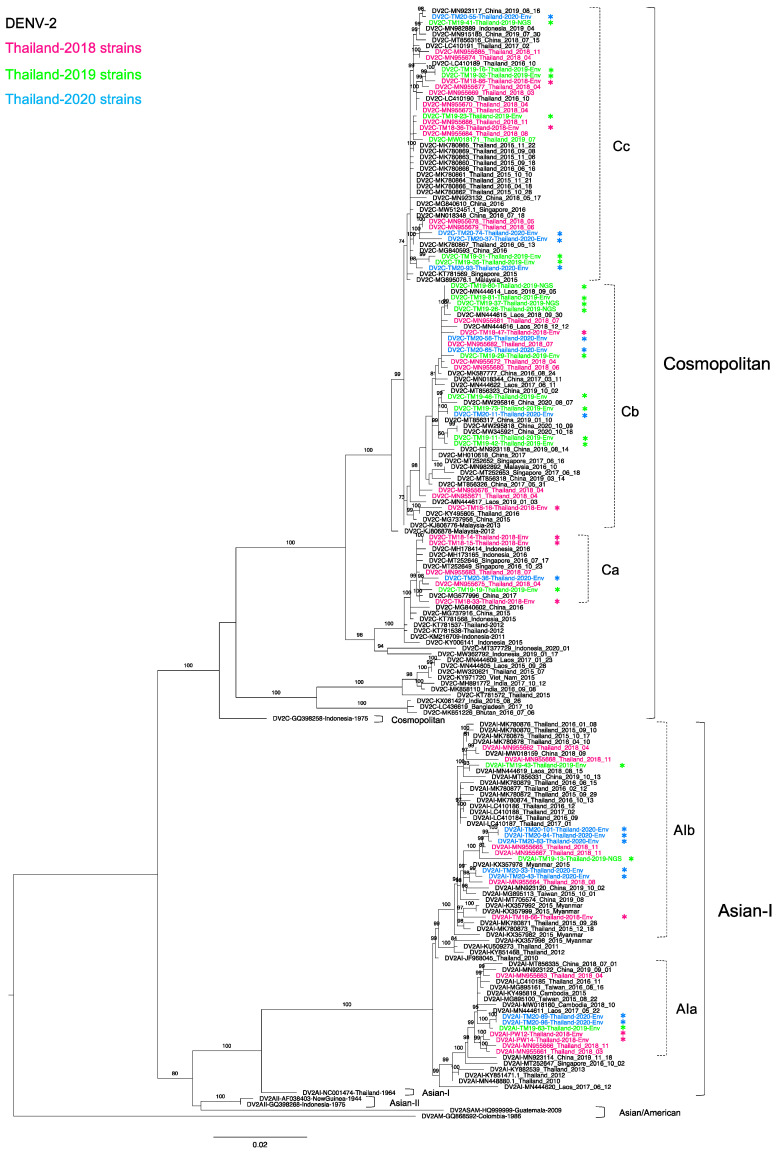
Phylogenetic analysis of Thailand DENV-2 in 2015–2020. The maximum-likelihood (ML) tree was constructed based on the complete envelope (E) region sequences (1485 nucleotides) under TIM2 + F + I + G4 with 1000 ultrafast bootstrap replicates. The ML tree included 44 newly determined sequences of DENV-2 from clinical specimens from Bangkok in 2018–2020, their related strains, Thailand strains during 2015–2020, and the reference genotypes. The sequences collected in 2018, 2019, and 2020 are labeled in pink, green, and blue, respectively, and indicated in the upper left corner. The sequences newly determined in the present study are marked with asterisks (*). Viral genotypes and clades are indicated by black and dotted brackets, respectively, on the right. Sequences are shown by serotype, accession number, country, and reported year. Numbers on branches are bootstrap support values exceeding 80%, except for clades Cb (73%) and Cc (74%).

Of 52 DENV-2-positive samples, we determined 44 complete envelope region nucleotide sequences. There were 31 genotype Cosmopolitan (DENV-2-C) and 13 genotype Asian I (DENV-2-AI) viruses, indicating co-circulation of these two genotypes. To investigate the epidemiologic occurrence of these two genotypes, we performed a phylogenetic analysis of strains related to our newly obtained sequences and recent Thailand strains in 2015–2020 (Figure 2). Our 13 DENV-2-AI strains formed two clades together with sequences from Thailand in 2016 and 2018 [30,32,52]. The five sequences in clade a (AIa) also clustered with strains from Laos, Cambodia in 2015-2017, and from Taiwanese travelers returning from Southeast Asia in 2015–2016 (MG895100 and MG895161) [49]. 

In contrast, eight remaining sequences were in clade b (AIb), which was larger than AIa. AIb consisted of Thailand strains in 2015-2020 from several parts of Thailand, particularly southern Thailand and Bangkok [35], Laos in 2018, Myanmar in 2015 [53], China in 2019, and travelers from Taiwan and China in Thailand or Southeast Asia [49,54]. Notably, both AIa and AIb viruses had the ancestral strain of the Thailand 2010 strains [48,52]. It is likely that DENV-2-AI has been circulating nearly continuously in Thailand from its first report in Thailand in 1964 until the present day.

All 31 DENV-2-C viruses belonged to lineage C, which includes viruses circulating primarily in Southeast Asia [55,56,57,58,59] and that share a high degree of similarity with sequences from Thailand in 2015–2018 [30,32]. We determined 7, 16, and 8 DENV-2-C sequences of viruses in 2018, 2019, and 2020, respectively. We combined these sequences with 18 sequences from 2018 in our previous study [30] and one 2019 sequence of Thailand DENV-2-C in the NCBI database. These DENV-2-C viruses formed three phylogenetic clades. DENV-2-C clade a (Ca) was a small clade, containing five of our new sequences in 2018–2020 along with strains from Indonesia, Singapore, and China collected in 2015–2017 [60,61]. This clade shared the ancestral strains of Thailand 2012 (KT781537, KT781538) [62] and Indonesia 2011 (KM216709) [63]. Within DENV-2-C clade b (Cb), our 14 strains newly obtained in 2018–2020 clustered with strains from Laos, China, and Singapore collected in 2017-2019 [64]. DENV-2-C clade c (Cc) contained half of the 2018 Thailand strains, which clustered with strains from southern Thailand [35], China, and Singapore collected in 2015–2019. In addition, DENV-2 clades Cb and Cc included a Malaysia strain from 2012 [65] (KJ806878) as a common ancestor.

### 3.4. Phylogenetic Analysis of Thailand DENV-3 Collected in 2015–2020

**Figure 3:**
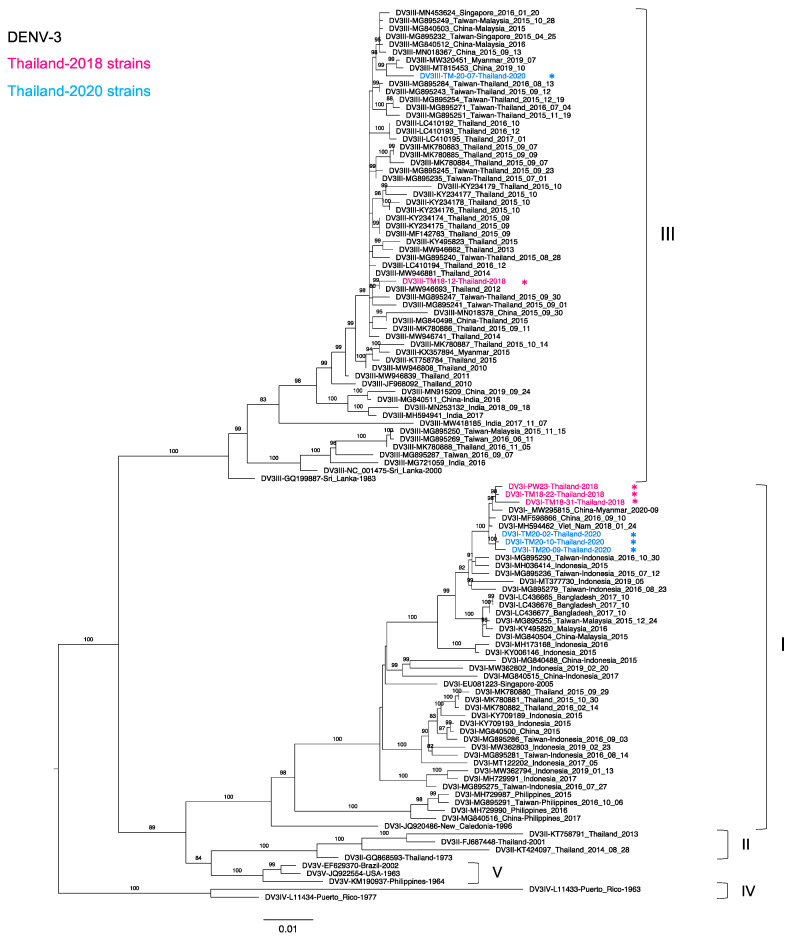
Phylogenetic analysis of Thailand DENV-3 in 2015–2020. The maximum-likelihood (ML) tree was constructed based on the complete envelope (E) region sequences (1479 nucleotides) under TN + F + I + G4 with 1000 ultrafast bootstrap replicates. The ML tree included eight newly determined sequences of DENV-3 from clinical specimens in Bangkok in 2018–2020, their related strains, Thailand strains during 2015–2020, and the reference genotypes. The sequences collected in 2018 and 2020 are labeled in pink and blue, respectively, and indicated in the upper left corner. Viral genotypes are indicated by black brackets on the right. The sequences newly determined in the present study are marked with asterisks (*). Sequences are shown by serotype, accession number, country, and reported year. Numbers on branches are bootstrap support values exceeding 80%.

We analyzed DENV-3 strains collected in Thailand in 2015–2020. A total of 26, 7, and 1 strains were found in the NCBI database in 2015, 2016, and 2017, respectively. Twenty-four Thailand 2015 strains were genotype III (DENV-3-III) as the major genotype, whereas two remaining strains were genotype I (DENV-3-I) collected from the southern part of Thailand. Although DENV-3 was rarely detected in the present study, we also detected the co-circulation of DENV-3-III and DENV-3-I in Thailand. Remarkably, six strains were identified as DENV-3-I, and the two remaining strains were identified as DENV-3-III. In the phylogenetic tree (Figure 3), our DENV-3-I strains collected in 2018 were apparently related to the strain detected along the China–Myanmar border in 2020 (MW295815). These sequences were clustered together with our Thailand 2020 strains and Vietnam 2018 (MH594462) and China 2016 (MF598866) strains. Interestingly, our newly determined Thailand DENV-3-I sequences were more distantly related to the southern Thailand 2015 [35] cluster than to our previous sequences from the Bangladesh 2017 [31] cluster. In contrast, our new DENV-3-III strains obtained in 2018 and 2020 were clustered with other Thailand strains and strains from Myanmar, China, and the travelers who came to Thailand [49]. In addition, all of the Thailand DENV-3-III strains that circulated in 2015–2020 descended from Thailand strains from 2010–2011 (JF968092 and MW946839).

### 3.5. Phylogenetic Analysis of Thailand DENV-4 in 2015–2020

**Figure 4:**
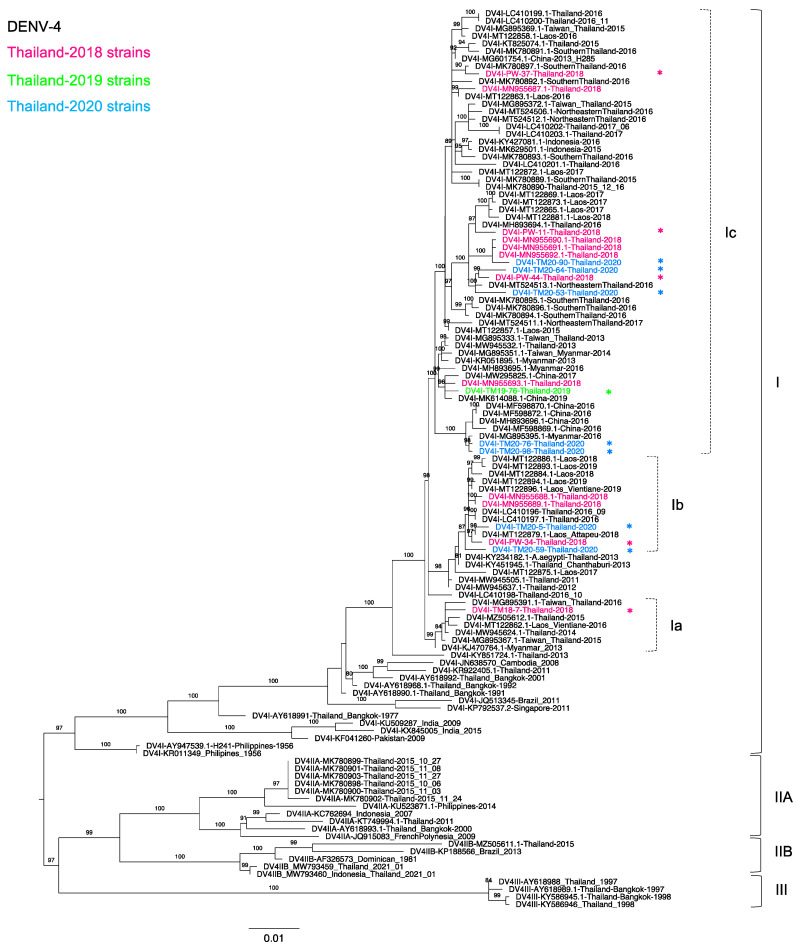
Phylogenetic analysis of Thailand DENV-4 in 2015–2020. The maximum-likelihood (ML) tree was constructed based on the complete envelope (E) gene sequences (1485 nucleotides) under TIM2 + F + G4 with 1000 ultrafast bootstrap replicates. The ML tree included 13 newly determined sequences of DENV-4 from clinical specimens from Bangkok in 2018–2020, their related strains, Thailand strains during 2015–2020, and the reference genotypes. The sequences collected in 2018, 2019, and 2020 are labeled in pink, green, and blue, respectively, and indicated in the upper left corner. The sequences newly determined in the present study are marked with asterisks (*). Viral genotypes and clades are indicated by black and dotted brackets, respectively, on the right. Sequences are shown by serotype, accession number, country, and reported year. Numbers on branches are bootstrap support values exceeding 80%.

Of 18 DENV-4 positive samples, we determined 13 sequences of the complete envelope region, including five from 2018, one from 2019, and seven from 2020. Figure 4 shows that all of our new sequences were classified as genotype I (DENV-4-I) lineage C, similar to our previous study [30], and these new sequences were related to the viruses recently reported in Thailand [30] and Southeast Asia, particularly in Laos, Myanmar, and Indonesia. Although only DENV-4-I predominated in the present study, three distinct clades were observed. TM18-7 was solely in a clade a (DENV-4-Ia), which contained sequences from Taiwanese patients with previous history of travel to Thailand in 2015 and 2016 [49] (MG895367 and MG895391), together with Thai strains collected in 2014–2015. These sequences shared the common ancestor of a Myanmar strain collected in 2013 (KJ470764). 

In contrast, PW-34, TM20-05, and TM20-59 were in clade b (DENV-4-Ib) and related to sequences from Thailand and Attapeu Province, Laos [30,32,66], collected on dates similar to our sequences collected in 2018–2019. Viruses isolated in Thailand in 2011–2012 (MW94550 and MW945637) were the ancestral strains of this clade. Clade c contained viruses from several parts of Thailand, Myanmar, Laos, China, and Indonesia. Thailand viruses in clade c exhibited substantial diversity, as TM19-76, TM20-76, and TM20-98 were closely related to Myanmar [49] and China strains, whereas PW-11, PW-44, TM20-53, TM20-64, and TM20-90 were closely related to strains from southern and northeastern Thailand collected in 2016 [35]. Furthermore, the phylogenetic tree indicated that our recent DENV-4-I strains were related to the previous strains that circulated in 1991 [27]. It is likely that DENV-4-I has been circulating in Thailand from its first detection in Thailand in 1977 until the present day. 

## 4. Discussion

Our present results reveal the molecular characteristics of DENV collected from suspected cases of dengue at the Hospital for Tropical Diseases, Bangkok, Thailand, during the period of 2018–2020. Either NGS or Sanger sequencing was used to reveal genetic variations in the envelope region of DENV gene. The NGS MiSeq platform (Illumina) was used to analyze patient serum samples with the highest viral load that could show minor genetic mutations, whereas Sanger sequencing was used for the remaining serum samples. We observed co-circulation of all four serotypes of DENV, with multiple genotypes and clades. The incidence and co-circulation of different DENVs affect the epidemic cycle of serotypes, genotypes or clade replacement, and inter-serotypic immune reactions [67,68]. Infection with related but distinct DENVs can cause antibody-dependent enhancement, which can lead to higher risks for manifestation of DHF and DSS [69,70]. Moreover, host immunity drives viral adaptation and evolution, including mutational drift and clade or genotype replacement [50,67]. DENV-1 was the dominant serotype in the present study, consistent with Thailand MoPH reports [11,12,71,72] indicating an increase in DENV-1 and decrease in DENV-3 and DENV-4 in 2019. Although the dominant DENV serotype in Thailand changed frequently during the period of 2005–2019, our study found that the dominant serotype was consistently DENV-1 in 2018–2020. All the DENV-1 obtained in the present study were genotype I. In Thailand, DENV-1 genotype I was first reported in 1981 and has been continuously detected in Thailand and Southeast Asia since that time [45,46,47,48,49,50,51]. Other genotypes have been reported only sporadically since genotype II was isolated in 1964 and have now disappeared. Genotype IV was found only in mosquitos in 2015, and genotype V was reported only in 1980 and 2015 [23,46,62,73,74]. Therefore, our results confirmed that DENV-1 genotype I continues to circulate in Thailand, consistent with results of previous studies in Thailand [30,32,35]. In addition, we observed four distinct clades of DENV-1 genotype I, indicating substantial levels of diversity among genotype I DENV-1 strains in Bangkok. The clade b viruses were similar to or related to strains collected in Thailand and neighboring countries, indicating a close genetic link between DENV-1 in several countries in Southeast Asia [45,53,75,76]. Moreover, the similarity between our sequences and those of viruses collected from China [48,49] suggests geographic expansion of DENV strains has occurred from Southeast Asia to east Asian countries through travelers and/or migration. 

Our DENV-2 sequences exhibited a genetic relationship with other DENV-2 strains isolated from geographically diverse areas, possibly due to globalization of the human population and migration of mosquito vectors [77]. The phylogenetic analysis of DENV-2 revealed co-circulation of two genotypes in Bangkok: Asian I and Cosmopolitan. Moreover, we observed more Cosmopolitan viruses than Asian I viruses, consistent with our previous study in 2018 [30,32]. Notably, our sequences distributed together with sequences from China in the three clades of the Cosmopolitan genotype and spread to various countries. The results of the present study suggested that the Cosmopolitan genotype originated from the Indonesian strains in 1975, spread to Malaysia or Thailand, and has since played a major role in DENV-2 distribution in Southeast Asia and Asia [78]. 

Among genotype Asian I viruses, we observed two distinct clades, which were related to two distinct Thailand 2010 strains. Asian I clade a contained viruses from Laos and Cambodia, and Asian I clade b contained viruses from Laos and Myanmar, suggesting that Bangkok and Thailand strains may have served as an epicenter for viral spread to Myanmar and Laos [79]. The phylogenetic analysis also revealed parallel dispersal of multiple genotypes and clades of DENV-2, suggesting that various introduced strains distributed separately in the same area. These variations may enable the virus to “hide” from the host immune response and lead to the circulation of a heterogenous virus population. Mosquitoes may maintain DENV genetic diversity, and adaptive processes and mosquito vectors can drive major DENV genotype or clade replacement [20]. 

In Thailand, DENV-3 was the predominant serotype in 2013–2015 [80], whereas DENV-3 was rarely detected in the present study and not detected at all in our previous 2018 study [30,32]. In the present study, we detected more genotype I than genotype III viruses. DENV-3 genotype I first emerged in Thailand in 1998 and re-appeared in the southern part of Thailand in 2015 [35]. Our new DENV-3 genotype I viruses detected in the capital city of Thailand in 2018 and 2020 formed a cluster distinct from those viruses in the southern part of Thailand, suggesting that the 2018 and 2020 viruses may have originated from similar sources and then spread to Bangkok. Furthermore, our sequences were clustered distinctly from the cluster of the viruses from the southern part of Thailand, indicating different origins of transmission. Notably, these sequences were in the same cluster as sequences from Taiwanese travelers to Indonesia, Singapore, and Malaysia, suggesting that DENV-3 genotype I continues to circulate and is predominant in these regions and neighboring countries. It will be necessary to carefully observe whether the re-appeared genotype I of DENV-3 becomes dominant in Bangkok in the future. 

The present study confirmed the re-occurrence and general circulation of DENV-3 genotype I with beneficial extinction of genotype II in urban regions. This genotype re-placement and extinction may reflect competition between genotypes with regard to different vectors and transmission patterns in the human population [21]. As our participants were enrolled in Bangkok, an urban region, further studies covering other parts of Thailand should be conducted to confirm the extinction of genotype II in this country. On the other hand, DENV-3 genotype III had predominated since 2010 but was only sporadically reported after 2017 [49]. Our new DENV-3 genotype III sequences were highly similar to those of viruses obtained in 2016 [32], suggesting infrequent but continuous spread of DENV-3 genotype III in Thailand, especially in Bangkok.

In the past, genotypes I, IIA, and III of DENV-4 co-circulated in Bangkok, whereas genotype I was first identified in 1977, after which it was continuously detected and has become the predominant genotype according to the present study [27,30,35]. Although all our sequences were genotype I, they clustered in three different clades. The phylogenetic tree suggested that Thailand is the center of DENV-4 distribution in Southeast Asia. Although genotype I emerged and spread through in Laos [66], these sequences originated from Thailand viruses showing a specific distribution in this area [81,82]. Moreover, DENV-4 dominated for only a short period in Thailand during 1993–1994 perhaps due to immunological cross-reaction between DENV-4 and the other serotypes in the population [66].

More than five decades have passed since the first case of DENV infection in Bangkok. Our present study during 2018–2020 in Bangkok revealed continuous co-circulation of four serotypes of DENVs and various distinct genotypes and clades in each serotype. Although previous data on DENV genetic diversity are limited, we speculate that the genetic diversity of DENV in Thailand has increased considerably over time due to increases in travel and globalization.

## 5. Conclusions

The present study demonstrated the co-circulation of all four serotypes of DENV in Bangkok, the capital city of Thailand, in 2018–2020. A high level of genetic diversity of DENV in this area was suggested by the presence of distinct genotypes of DENV-2 and DENV-3 and several different clades in a single genotype of DENV-1 and DENV-4. Continuous molecular monitoring of DENVs in Bangkok is thus warranted for better control of dengue and the development of effective vaccines in endemic areas.

## Figures and Tables

**Table 1:** The percentage of DENV serotype positive samples among participants from the Hospital for Tropical Diseases, Bangkok, Thailand, during 2018–2020.

Year	No. of DENV Suspected Patient	No. of Participants	% of Positive (No. of Positive)			
			DENV-1	DENV-2	DENV-3	DENV-4
2018	540	88	40.9% (36)	17.0% (15)	9.1% (8)	20.5% (18)
2019	424	81	44.4% (36)	27.2% (22)	0%	2.5% (2)
2020	136	107	45.8% (49)	14.0% (15)	3.7% (4)	11.2% (12)
Total	1100	276	43.8% (121)	18.8% (52)	4.3% (12)	11.6% (32)

## Data Availability

Dengue virus sequences were submitted to the GenBank database as described in Table S4. (Sequences are shown by serotype, accession number, country, and reported year in Figure 1, Figure 2, Figure 3 and Figure 4).

## Supplementary Materials

The following are available online at https://www.mdpi.com/article/10.3390/tropicalmed6030162/s1, Table S1: Primers used for whole-genome sequencing and sample preparation; Table S2: Primers used for sequencing of the complete envelope region and sample preparation; Table S3: Sequences of the envelope region obtained from GenBank used in phylogenetic analyses; Table S4: Characteristics of DENV samples analyzed in the present study from Hospital for Tropical Diseases, Bangkok, Thailand during 2018–2020.
