# Supplementary material for: Genetic Diversity of Dengue Virus in Clinical Specimens from Bangkok, Thailand, during 2018–2020: Co-Circulation of All Four Serotypes with Multiple Genotypes and/or Clades
Source: Trop Med Infect Dis. 2021 Sep 4;6(3):162. doi: 10.3390/tropicalmed6030162 (PMC8482112; doi:10.3390/tropicalmed6030162)
Supplement: Supplementary file 1 [file tropicalmed-06-00162-s001.zip › tropicalmed-1360157-supplementary.pdf]

Tropical Medicine and Infectious Disease

MDPI

Article

Genetic Diversity of Dengue Virus in Clinical Specimens from Bangkok, Thailand, during 2018–2020: Co-Circulation of All Four Serotypes with Multiple Genotypes and/or Clades

Kanaporn Poltep [1,2,3], Juthamas Phadungsombat [2,4], Emi E. Nakayama [2,4], Nathamon Kosoltanapiwat [1], Borimas Hanboonkunupakarn [5], Witthawat Wiriyarat [3], Tatsuo Shioda [2,4,*] and Pornsawan Leaungwutiwong [1,*]

[1] Department of Microbiology and Immunology, Faculty of Tropical Medicine, Mahidol University, Bangkok 10400, Thailand; kanaporn.pol@gmail.com (K.P.); nathamon.kos@mahidol.ac.th (N.K.)

[2] Mahidol-Osaka Center for Infectious Diseases (MOCID), Faculty of Tropical Medicine, Mahidol University, Bangkok 10400, Thailand; juthamas@biken.osaka-u.ac.jp (J.P.); emien@biken.osaka-u.ac.jp (E.E.N.)

[3] The Monitoring and Surveillance Center for Zoonotic Diseases in Wildlife and Exotic Animals, Faculty of Veterinary Science, Mahidol University, Nakhon Pathom, 73170, Thailand; witthawat.wir@mahidol.edu

[4] Department of Viral Infections, Research Institute for Microbial Diseases (RIMD), Osaka University, Osaka 565-0871, Japan

[5] Department of Clinical Tropical Medicine, Faculty of Tropical Medicine, Mahidol University, Bangkok 10400, Thailand; borimas.han@mahidol.ac.th

* Correspondence: shioda@biken.osaka-u.ac.jp (T.S.) and pornsawan.lea@mahidol.ac.th (P.L.)

Citation: Poltep, K.; Phadungsombat, J.; Nakayama, E.E.; Kosoltanapiwat, N.; Hanboonkunupakarn, B.; Wiriyarat, W.; Shioda, T.; Leaungwutiwong, P. Genetic Diversity of Dengue Virus in Clinical Specimens from Bangkok, Thailand, during 2018-2020: Co-Circulation of All Four Serotypes with Multiple Genotypes and/or Clades. *Trop. Med. Infect. Dis.* **2021**, *6*, 162. https://doi.org/10.3390/tropicalmed6030162

Academic Editor: John McBride

Received: 13 August 2021
Accepted: 1 September 2021
Published: 4 September 2021

Publisher's Note: MDPI stays neutral with regard to jurisdictional claims in published maps and institutional affiliations.

Copyright: © 2021 by the authors. Licensee MDPI, Basel, Switzerland. This article is an open access article distributed under the terms and conditions of the Creative Commons Attribution (CC BY) license (http://creativecommons.org/licenses/by/4.0/).

Supplementary

Table S1. Primers used for whole-genome sequencing and sample preparation.

Fragment	RT primer	Sequence 5'-3'	PCR primer	Sequence 5'-3'
DENV1-5	d1a10 [a]	TCTCTCYGGCTCRAAGAGGG	d1s1C [a]	*GATGAGGGAAGATGGGG*AGTTGTTAGTCTACGTGGAC
			d1a10 [a]	TCTCTCYGGCTCRAAGAGGG
DENV1-3	d1a5B [a]	*TTTGTCGGTCTGGGGGGGTAT*AGAACCTGTTGATTCAACRGC	d1s12 [a]	AAATGGCAGAGGCGCTCAAGGG
			d1a5B [a]	*TTTGTCGGTCTGGGGGGGTAT*AGAACCTGTTGATTCAACRGC
DENV2-5	DV2RT6817 [b]	TGCGGCCACCACTGTGAGGATGGC	T7-5TDVFwClal [b]	GGCATCGATTAATACGACTCACTATAGAGTTGTTAGTCTACGTGGACCGACAAAG
			DV2RvNhel6731Notl [b]	GGCGCGGCCGCAGCAAAACTATGAGAAAAAACTCCA
DENV2-3	3T DV Rv [b]	AGAACCTGTTGATTCAACAGCACC	DV2FwClal6608Nhel [b]	GGCATCGATAGGGAAGATGACCCTGGGAATGTG
			3T-Notl DV Rv [b]	GCCGCGGCCGCAGAACCTGTTGATTCAACAGCACC

Underlying represent restriction sites or T7 promoter sequences that are not present in dengue virus genomes. [a]Christenbury, J. G., 2010. [b]Kurosu, T., 2010.

Table S2. Primers used for complete envelope-regions sequencing and sample preparation.

**Fragment**		**Antisense-primers**	**Sequence 5'-3'**	**Sense-primers**	**Sequence 5'-3'**
DENV1-E	One-Step RT	d1a17[a]	CCAATGGCYGCTGAYAGTCT	d1s1C [a]	GATGAGGGAAGATGGGGAGTTGTTAGTCTACGTGGAC
	Nested PCR	d1a17[a]	CCAATGGCYGCTGAYAGTCT	d1s3 [a]	ATAAACGTTCTGTCGCATTGGC
	Sequencing	d1a17[a]	CCAATGGCYGCTGAYAGTCT	d1s3 [a]	ATAAACGTTCTGTCGCATTGGC
		1371R[c]	TCCAGTGTGGACGGTGACTA	d1s4 [a]	TGTGTGTCGACGAACATT
				d1s5 [a]	GCAATGCAYACTGCGTTG
DENV2-E	One-Step RT	2447R	TTCCAGCTCACAACGCAACCAC	d2s1C [a]	GATGAGGGAAGATGGGGAGTTGTTAGTCTACGTGGAC
	Nested PCR	2447R	TTCCAGCTCACAACGCAACCAC	618F	ACCAGAAGACATAGAYTGTTGGTG
	Sequencing	2447R	TTCCAGCTCACAACGCAACCAC	911F	TGACAGCTGTCGCTCCTTCAATG
		1631R[d]	ATACAGAARGARACATTGGTC	1577F	TAGACCTGCCATTACCATG
		1602R	CGGAGCAGACACACAAGGAT	d2seq4	RGGTGACACAGCCTGGGA
DENV3-E	One-Step RT	DV3R	CCACAACTACCGTTAATTTGAT	d3s1C [a]	GATGAGGGAAGATGGGGAGTTGTTAGTCTACGTGGAC
	Nested PCR	DV3R	CCACAACTACCGTTAATTTGAT	815F [e]	GCCCTTAGGCACCCAGGGTT
	Sequencing	DV3R	CCACAACTACCGTTAATTTGAT	815F [e]	GCCCTTAGGCACCCAGGGTT
				E2F [f]	CGGTTGTGGTTTGTTTGG
				Seq2C [f]	GCCATCTTGGGAGACA
				d3s4 [a]	GAAGAACAAAGCATGGATGGTA
				d3s5 [a]	TGAACCTCCTTTTGGGGAA
				1977F	GACTGATCACAGCCAACCCA
Fragment		Antisense-primers	Sequence 5'-3'	Sense-primers	Sequence 5'-3'
DENV4-E	One-Step RT	d4a18 [a]	GGGCATTYAATATTGCAGACGCTA	D41F	AGTTGTTAGTCTACGTGGACCGACAA
	Nested PCR	d4a18 [a]	GGGCATTYAATATTGCAGACGCTA	d4EF1	CTAATGATGCTGGTCGCCC
	Sequencing	d4a18 [a]	GGGCATTYAATATTGCAGACGCTA	d4EF1	CTAATGATGCTGGTCGCCC
		d4a6 [a]	TGGGTGTCTCCATTGTGGACTG	d4s3 [a]	TTTGAACTGACYAAGACAACAGC
				d4s4 [a]	TGGACAGCAGGAGCAGACACAT
				d4s6 [a]	GYTCCATTGGCAAGATGTTTGAG

Underlying represent restriction sites or T7 promoter sequences that are not present in dengue virus genomes. [a] Christenbury, J. G., 2010. [b] Kurosu, T., 2010. [c] Hamel, R., 2019. [d] Singh, S., 2015. [e] Phommanivong, V., 2016. [f] Luo, L., 2013.

Table S3. Sequences of the envelope region from GenBank used in phylogenetic analysis.

Serotype	Genotype	Accession Number	Country	Year
DENV-1	I	AB074760.1	Japan	1943
DENV-1	I	AF350498.1	China	1980
DENV-1	I	AY732481.1	Thailand	1982
DENV-1	I	AY732477.1	Thailand	1991
DENV-1	I	AY732475.1	Thailand	1994
DENV-1	I	AY732479.1	Thailand	2001
DENV-1	I	AY732482.1	Thailand	2001
DENV-1	I	AY732480.1	Thailand	2004
DENV-1	I	HG316481.1	Thailand	2010
DENV-1	I	KX595191.1	Vietnam	2013
DENV-1	I	KF887994.1	Thailand	2013
DENV-1	I	LC002828.1	Japan	2014
DENV-1	I	MK780858.1	Thailand, Southern	2016
DENV-1	I	MK780859.1	Thailand, Southern	2016
DENV-1	I	LC410183.1	Thailand	2017
DENV-1	I	MN955621.1	Thailand	2018
DENV-1	I	MN955622.1	Thailand	2018
DENV-1	I	MN955623.1	Thailand	2018
DENV-1	I	MN955625.1	Thailand	2018
DENV-1	I	MN955629.1	Thailand	2018
DENV-1	I	MN955631.1	Thailand	2018
DENV-1	I	MN955646.1	Thailand	2018
DENV-1	I	MN955648.1	Thailand	2018
DENV-1	I	MN955650.1	Thailand	2018
DENV-1	I	MN955653.1	Thailand	2018
DENV-1	I	MN955657.1	Thailand	2018
DENV-1	II	AF180817.1	Thailand	1964
DENV-1	III	EF457905.1	Malaysia	1972
DENV-1	IV	AB189120.1	Indonesia	1998
DENV-1	IV	AY722803.1	Myanmar	1998
DENV-1	IV	DQ672562.1	USA	2001
DENV-1	IV	DQ285560.1	Reunion	2004
DENV-1	IV	GQ868602.1	Philippines	2004
DENV-1	IV	KT827366.1	China	2007
DENV-1	IV	KC762651.1	Indonesia	2007
DENV-1	V	AY732474.1	Thailand	1980
DENV-1	V	AF226685.1	Brazil	1990
DENV-1	V	AF514889.1	Argentina	2000
DENV-1	V	KF289072.1	India	2011
DENV-1	VI	KR919820.1	Brunei	2014
DENV-1	VI	KT825058.1	Thailand	2013
DENV-1	I	MW946024.1	Thailand	1995
DENV-1	I	AY732459.1	Thailand	1997
DENV-1	I	KY586442.1	Thailand	2003
DENV-1	I	MW945811.1	Thailand	2005
DENV-1	I	KY586429.1	Thailand, Bangkok	2006
Serotype	Genotype	Accession Number	Country	Year

DENV-1	I	JF967810.1	Myanmar	2008
DENV-1	I	JF967796.1	Malaysia	2008
DENV-1	I	HQ591537.1	Vietnam	2008
DENV-1	I	LC148025.1	Cambodia	2008
DENV-1	I	JF960220.1	Singapore	2010
DENV-1	I	MG564072.1	Thailand	2011
DENV-1	I	JQ048541.1	China	2011
DENV-1	I	LC148030.1	Maldives	2011
DENV-1	I	MF033202.1	Singapore	2012
DENV-1	I	KY818153.1	Malaysia	2012
DENV-1	I	KR527488.1	India	2012
DENV-1	I	KT825058.1	Thailand	2013
DENV-1	I	MG894763.1	Taiwan, Thai	2013
DENV-1	I	MW945810.1	Thailand	2013
DENV-1	I	MW945883.1	Thailand	2013
DENV-1	I	MW945998.1	Thailand	2013
DENV-1	I	MW946242.1	Thailand	2013
DENV-1	I	KU509291.1	German, Thai	2013
DENV-1	I	KJ806855.1	Malaysia	2013
DENV-1	I	KJ806941.2	Singapore	2013
DENV-1	I	KJ806946.2	Singapore	2013
DENV-1	I	KJ806952.1	Singapore	2013
DENV-1	I	KR051920.1	Myanmar	2013
DENV-1	I	MW945942.1	Thailand	2014
DENV-1	I	KU509313.1	German, Singapore	2014
DENV-1	I	MG894818.1	Taiwan, Myanmar	2014
DENV-1	I	MG737900.1	China	2015
DENV-1	I	MF033239.1	Singapore	2015
DENV-1	I	KX357940.2	Myanmar	2015
DENV-1	I	MW302772.1	Myanmar	2015
DENV-1	I	KY672936.1	China, Yunnan	2015
DENV-1	I	MG894863.1	Taiwan, Myanmar	2015
DENV-1	I	KT825039.1	Myanmar	2015
DENV-1	I	MG894893.1	Taiwan, Vietnam	2015
DENV-1	I	MF033260.1	Singapore	2016
DENV-1	I	MF033254.1	Singapore	2016
DENV-1	I	MW302778.1	China	2016
DENV-1	I	MG894965.1	China, Vietnam	2016
DENV-1	I	MH729967.1	Myanmar	2017
DENV-1	I	MG679801.1	Myanmar	2017
DENV-1	I	MG696656.1	Myanmar	2017
DENV-1	I	MG840577.1	China	2017
DENV-1	I	MH594890.1	India	2017
DENV-1	I	MK529748.1	China	2018
DENV-1	I	MW014049.1	Thailand	2018
DENV-1	I	MN933739.1	China	2018
DENV-1	I	MN921284.1	China	2019
DENV-1	I	MN921368.1	China, Cambodia	2019
DENV-1	I	MW228041.1	Cambodia	2019
DENV-1	I	MN921518.1	China, Cambodia	2019
DENV-1	I	MT705147.1	Myanmar	2019

Serotype	Genotype	Accession Number	Country	Year
DENV-1	I	MT705153.1	Myanmar	2019
DENV-1	I	MT705151.1	Myanmar	2019
DENV-1	I	MN960677.1	China	2019
DENV-1	I	MN921258.1	China, Thailand	2019
DENV-1	I	MZ313995.1	India	2021
DENV-2	American	GQ868592	Cambodia	1986
DENV-2	Asian I	MN444620	Laos	2017
DENV-2	Asian I	MN955668	Thailand	2018
DENV-2	Asian I	KX357982	Myanmar	2015
DENV-2	Asian I	MN923120	China	2019
DENV-2	Asian I	MT856331	China	2019
DENV-2	Asian I	MN955665	Thailand	2018
DENV-2	Asian I	MN955667	Thailand	2018
DENV-2	Asian I	KX357992	Myanmar	2015
DENV-2	Asian I	KX357999	Myanmar	2015
DENV-2	Asian I	MT705574	China	2019
DENV-2	Asian I	MK780871	Thailand	2015
DENV-2	Asian I	MK780873	Thailand	2015
DENV-2	Asian I	MN955664	Thailand	2018
DENV-2	Asian I	MN955662	Thailand	2018
DENV-2	Asian I	MW018159	China	2018
DENV-2	Asian I	MN444619	Laos	2018
DENV-2	Asian I	MK780876	Thailand	2016
DENV-2	Asian I	MK780875	Thailand	2015
DENV-2	Asian I	MK780878	Thailand	2016
DENV-2	Asian I	MK780870	Thailand	2015
DENV-2	Asian I	MG895113	Taiwan	2015
DENV-2	Asian I	MK780879	Thailand	2016
DENV-2	Asian I	MK780872	Thailand	2015
DENV-2	Asian I	KX357978	Myanmar	2015
DENV-2	Asian I	MK780874	Thailand	2016
DENV-2	Asian I	LC410184	Thailand	2016
DENV-2	Asian I	MK780877	Thailand	2016
DENV-2	Asian I	LC410186	Thailand	2016
DENV-2	Asian I	LC410188	Thailand	2017
DENV-2	Asian I	LC410187	Thailand	2017
DENV-2	Asian I	MT252647	Singapore	2016
DENV-2	Asian I	KX357998	Myanmar	2015
DENV-2	Asian I	MN923114	China	2019
DENV-2	Asian I	MT856335	China	2018
DENV-2	Asian I	MW018160	Cambodia	2018
DENV-2	Asian I	MN923122	China	2019
DENV-2	Asian I	LC410185	Thailand	2016
DENV-2	Asian I	MN955663	Thailand	2018
DENV-2	Asian I	MN444611	Laos	2017
DENV-2	Asian I	MG895161	Taiwan	2016
DENV-2	Asian I	KY495819	Cambodia	2015
DENV-2	Asian I	MG895100	Taiwan	2015
DENV-2	Asian I	MN955666	Thailand	2018
DENV-2	Asian I	MN955661	Thailand	2018

DENV-2	Asian I	KU509273	Thailand	2011
Serotype	Genotype	Accession Number	Country	Year
DENV-2	Asian I	KY851468	Thailand	2012
DENV-2	Asian I	JF968045	Thailand	2010
DENV-2	Asian I	KY882539	Thailand	2013
DENV-2	Asian I	KY851471.1	Thailand	2012
DENV-2	Asian I	MN448880.1	Thailand	2010
DENV-2	Asian I	NC001474	Thailand	1964
DENV-2	Asian II	AF038403	New Guinea	1944
DENV-2	Asian II	GQ398268	Indonesia	1975
DENV-2	Asian/American	HQ999999	Guatemala	2009
DENV-2	Cosmopolitan	MK651226	Bhutan	2016
DENV-2	Cosmopolitan	KX061427	India	2015
DENV-2	Cosmopolitan	LC436619	Bangladesh	2017
DENV-2	Cosmopolitan	KT781572	Thailand	2015
DENV-2	Cosmopolitan	KY971720	Vietnam	2015
DENV-2	Cosmopolitan	MN444609	Laos	2017
DENV-2	Cosmopolitan	MN444605	Laos	2015
DENV-2	Cosmopolitan	MW320621	Thailand	2015
DENV-2	Cosmopolitan	MH891772	India	2017
DENV-2	Cosmopolitan	MK858110	India	2016
DENV-2	Cosmopolitan	MT856318	China	2019
DENV-2	Cosmopolitan	MN923118	China	2019
DENV-2	Cosmopolitan	MN982892	Malaysia	2016
DENV-2	Cosmopolitan	MT252653	Singapore	2017
DENV-2	Cosmopolitan	MN955676	Thailand	2018
DENV-2	Cosmopolitan	MN955671	Thailand	2018
DENV-2	Cosmopolitan	MN444616	Laos	2018
DENV-2	Cosmopolitan	MW295816	China	2020
DENV-2	Cosmopolitan	MN018344	China	2017
DENV-2	Cosmopolitan	MN444622	Laos	2017
DENV-2	Cosmopolitan	MT856326	China	2017
DENV-2	Cosmopolitan	MN444615	Laos	2018
DENV-2	Cosmopolitan	MK587777	China	2016
DENV-2	Cosmopolitan	MN955680	Thailand	2018
DENV-2	Cosmopolitan	MN955681	Thailand	2018
DENV-2	Cosmopolitan	MN444614	Laos	2018
DENV-2	Cosmopolitan	MN955682	Thailand	2018
DENV-2	Cosmopolitan	MN955672	Thailand	2018
DENV-2	Cosmopolitan	MW295818	China	2020
DENV-2	Cosmopolitan	MW345921	China	2020
DENV-2	Cosmopolitan	MT856317	China	2019
DENV-2	Cosmopolitan	MT856323	China	2019
DENV-2	Cosmopolitan	MT252652	Singapore	2017
DENV-2	Cosmopolitan	MH010618	China	2017
DENV-2	Cosmopolitan	KJ806878	Malaysia	2012
DENV-2	Cosmopolitan	MN444617	Laos	2019
DENV-2	Cosmopolitan	MN923132	China	2018
DENV-2	Cosmopolitan	LC410189	Thailand	2016
DENV-2	Cosmopolitan	KY495805	Thailand	2016
DENV-2	Cosmopolitan	MG737956	China	2015

DENV-2	Cosmopolitan	MN955677	Thailand	2018
DENV-2	Cosmopolitan	MT856316	China	2018
Serotype	Genotype	Accession Number	Country	Year
DENV-2	Cosmopolitan	MN955678	Thailand	2018
DENV-2	Cosmopolitan	MN955679	Thailand	2018
DENV-2	Cosmopolitan	LC410191	Thailand	2017
DENV-2	Cosmopolitan	MN915185	China	2019
DENV-2	Cosmopolitan	MN923117	China	2019
DENV-2	Cosmopolitan	MN982889	Indonesia	2019
DENV-2	Cosmopolitan	KT781569	Singapore	2015
DENV-2	Cosmopolitan	MN955685	Thailand	2018
DENV-2	Cosmopolitan	MW018171	Thailand	2019
DENV-2	Cosmopolitan	MN955669	Thailand	2018
DENV-2	Cosmopolitan	MN955670	Thailand	2018
DENV-2	Cosmopolitan	MN955673	Thailand	2018
DENV-2	Cosmopolitan	LC410190	Thailand	2016
DENV-2	Cosmopolitan	KJ806776	Malaysia	2013
DENV-2	Cosmopolitan	MG895076.1	Malaysia	2015
DENV-2	Cosmopolitan	MK780867	Thailand	2016
DENV-2	Cosmopolitan	MN955674	Thailand	2018
DENV-2	Cosmopolitan	MN955684	Thailand	2018
DENV-2	Cosmopolitan	MN955686	Thailand	2018
DENV-2	Cosmopolitan	MK780860	Thailand	2015
DENV-2	Cosmopolitan	MK780863	Thailand	2015
DENV-2	Cosmopolitan	MK780868	Thailand	2016
DENV-2	Cosmopolitan	MK780865	Thailand	2015
DENV-2	Cosmopolitan	MK780869	Thailand	2016
DENV-2	Cosmopolitan	MW512451.1	Singapore	2016
DENV-2	Cosmopolitan	MG840593	China	2016
DENV-2	Cosmopolitan	MG840610	China	2016
DENV-2	Cosmopolitan	MK780861	Thailand	2015
DENV-2	Cosmopolitan	MK780862	Thailand	2015
DENV-2	Cosmopolitan	MK780864	Thailand	2015
DENV-2	Cosmopolitan	MK780866	Thailand	2016
DENV-2	Cosmopolitan	MN018348	China	2016
DENV-2	Cosmopolitan	MT377729	Indonesia	2020
DENV-2	Cosmopolitan	MW362792	Indonesia	2019
DENV-2	Cosmopolitan	MG840602	China	2016
DENV-2	Cosmopolitan	KY006141	Indonesia	2015
DENV-2	Cosmopolitan	MN955675	Thailand	2018
DENV-2	Cosmopolitan	MH178414	Indonesia	2016
DENV-2	Cosmopolitan	MG677996	China	2017
DENV-2	Cosmopolitan	MH173165	Indonesia	2016
DENV-2	Cosmopolitan	MT252646	Singapore	2016
DENV-2	Cosmopolitan	MN955683	Thailand	2018
DENV-2	Cosmopolitan	MT252649	Singapore	2016
DENV-2	Cosmopolitan	KT781537	Thailand	2012
DENV-2	Cosmopolitan	KT781538	Thailand	2012
DENV-2	Cosmopolitan	KM216709	Indonesia	2011
DENV-2	Cosmopolitan	MG737916	China	2015
DENV-2	Cosmopolitan	KT781568	Indonesia	2015

Serotype	Genotype	Accession Number	Country	Year
DENV-2	Cosmopolitan	GQ398258	Indonesia	1975
DENV-3	I	MG840516	China, Philippines	2017
DENV-3	I	MH729990	Philippines	2016
DENV-3	I	MH729987	Philippines	2015
DENV-3	I	MG895291	Taiwan, Philippines	2016
DENV-3	I	JQ920486	New Caledonia	1996
DENV-3	I	MH173168	Indonesia	2016
DENV-3	I	KY006146	Indonesia	2015
DENV-3	I	LC436665	Bangladesh	2017
DENV-3	I	LC436676	Bangladesh	2017
DENV-3	I	LC436677	Bangladesh	2017
DENV-3	I	MG840504	China, Malaysia	2015
DENV-3	I	MG895255	Taiwan, Malaysia	2015
DENV-3	I	KY495820	Malaysia	2016
DENV-3	I	MT377730	Indonesia	2019
DENV-3	I	MG895279	Taiwan, Indonesia	2016
DENV-3	I	MG895236	Taiwan, Indonesia	2015
DENV-3	I	MW295815	China, Myanmar	2020
DENV-3	I	MF598866	China	2016
DENV-3	I	MH594462	Vietnam	2018
DENV-3	I	MG895290	Taiwan, Indonesia	2016
DENV-3	I	MH036414	Indonesia	2015
DENV-3	I	MG840488	China, Indonesia	2015
DENV-3	I	MG840515	China, Indonesia	2017
DENV-3	I	MW362802	Indonesia	2019
DENV-3	I	EU081223	Singapore	2005
DENV-3	I	MG895275	Taiwan, Indonesia	2016
DENV-3	I	MW362794	Indonesia	2019
DENV-3	I	MH729991	Indonesia	2017
DENV-3	I	MT122202	Indonesia	2017
DENV-3	I	MW362803	Indonesia	2019
DENV-3	I	MG895281	Taiwan, Indonesia	2016
DENV-3	I	KY709189	Indonesia	2015
DENV-3	I	MK780880	Thailand	2015
DENV-3	I	MK780881	Thailand	2015
DENV-3	I	MK780882	Thailand	2016
DENV-3	I	KY709193	Indonesia	2015
DENV-3	I	MG840500	China	2015
DENV-3	I	MG895286	Taiwan, Indonesia	2016
DENV-3	II	KT424097	Thailand	2014
DENV-3	II	KT758791	Thailand	2013
DENV-3	II	FJ687448	Thailand	2001
DENV-3	II	GQ868593	Thailand	1973
DENV-3	III	MW418185	India	2017
DENV-3	III	MN915209	China	2019
DENV-3	III	MG840511	China, Indonesia	2017
DENV-3	III	MN253132	India	2018
DENV-3	III	MH594941	India	2017
DENV-3	III	KY234179	Thailand	2015
DENV-3	III	MN018378	China	2015

Serotype	Genotype	Accession Number	Country	Year
DENV-3	III	JF968092	Thailand	2010
DENV-3	III	MK780887	Thailand	2015
DENV-3	III	KX357894	Myanmar	2015
DENV-3	III	MW946839	Thailand	2011
DENV-3	III	MW946808	Thailand	2010
DENV-3	III	KT758784	Thailand	2015
DENV-3	III	KY234177	Thailand	2015
DENV-3	III	MG895251	Taiwan, Thailand	2015
DENV-3	III	MG895254	Taiwan, Thailand	2015
DENV-3	III	MG895271	Taiwan, Thailand	2016
DENV-3	III	MW320451	Myanmar	2019
DENV-3	III	MT815453	China	2019
DENV-3	III	KY495823	Thailand	2015
DENV-3	III	MG895241	Taiwan, Thailand	2015
DENV-3	III	MK780884	Thailand	2015
DENV-3	III	MK780883	Thailand	2015
DENV-3	III	MK780885	Thailand	2015
DENV-3	III	MW946662	Thailand	2013
DENV-3	III	KY234178	Thailand	2015
DENV-3	III	KY234176	Thailand	2015
DENV-3	III	LC410195	Thailand	2017
DENV-3	III	LC410192	Thailand	2016
DENV-3	III	LC410193	Thailand	2016
DENV-3	III	MG895247	Taiwan, Thailand	2015
DENV-3	III	MG895240	Taiwan, Thailand	2015
DENV-3	III	MK780886	Thailand	2015
DENV-3	III	MG840498	China, Thailand	2015
DENV-3	III	MN453624	Singapore	2016
DENV-3	III	MN018367	China	2015
DENV-3	III	MG840512	China, Malaysia	2016
DENV-3	III	MG840503	China, Malaysia	2015
DENV-3	III	MG895249	China, Malaysia	2015
DENV-3	III	MG895232	Taiwan, Singapore	2015
DENV-3	III	MG895284	Taiwan, Thailand	2016
DENV-3	III	MG895243	Taiwan, Thailand	2015
DENV-3	III	KY234174	Thailand	2015
DENV-3	III	MF142763	Thailand	2015
DENV-3	III	KY234175	Thailand	2015
DENV-3	III	MW946741	Thailand	2014
DENV-3	III	MW946693	Thailand	2012
DENV-3	III	LC410194	Thailand	2016
DENV-3	III	MW946881	Thailand	2014
DENV-3	III	MG895245	Taiwan, Thailand	2015
DENV-3	III	MG895235	Taiwan, Thailand	2015
DENV-3	III	GQ199887	Sri Lanka	1983
DENV-3	III	NC001475	Sri Lanka	2000
DENV-3	III	MG721059	India	2016
DENV-3	III	MG895287	Taiwan	2016
DENV-3	III	MG895250	Taiwan, Malaysia	2015
DENV-3	III	MG895269	Taiwan	2016

DENV-3	III	MK780888	Thailand	2016
DENV-3	IV	L11433	Puerto Rico	1963
DENV-3	IV	L11434	Puerto Rico	1977
DENV-3	V	KM190937	Philippines	1964
DENV-3	V	EF629370	Brazil	2002
Serotype	Genotype	Accession Number	Country	Year
DENV-3	V	JQ922554	USA	1963
DENV-4	I	AY947539.1	Philippines	1956
DENV-4	I	KR011349	Philippines	1956
DENV-4	I	AY618991	Thailand_Bangkok	1977
DENV-4	I	FJ196850	China	1990
DENV-4	I	AY618990.1	Thailand_Bangkok	1991
DENV-4	I	AY618968.1	Thailand_Bangkok	1992
DENV-4	I	AY618992	Thailand_Bangkok	2001
DENV-4	I	MW945732.1	Thailand	2006
DENV-4	I	JN638570	Cambodia	2008
DENV-4	I	KF041260	Pakistan	2009
DENV-4	I	KU509287	India	2009
DENV-4	I	MW945645.1	Thailand	2010
DENV-4	I	MW945505.1	Thailand	2011
DENV-4	I	JQ513345	Brazil	2011
DENV-4	I	KP792537.2	Singapore	2011
DENV-4	I	KR922405.1	Thailand	2011
DENV-4	I	MN449005.1	Thailand	2012
DENV-4	I	MW945637.1	Thailand	2012
DENV-4	I	MG601754.1	China	2013
DENV-4	I	KJ470764.1	Myanmar	2013
DENV-4	I	KJ470765.1	Myanmar	2013
DENV-4	I	MK780900.1	Southern Thailand	2015
DENV-4	I	KX845005	India	2015
DENV-4	I	KX845005	India	2015
DENV-4	I	MK780894.1	Southern Thailand	2016
DENV-4	I	LC410197.1	Thailand	2016
DENV-4	II	NC_002640		
DENV-4	IIA	JQ915083	French Polynesia	2009
DENV-4	IIA	KC762694	Indonesia	2007
DENV-4	IIA	KU523871.1	Philippines	2014
DENV-4	IIB	FJ639773	Venezuela	2001
DENV-4	IIB	AF326573	Dominican	1981
DENV-4	IIB	AY618993.1	Thailand_Bangkok	2000
DENV-4	IIB	FJ850059	Puerto Rico	1998
DENV-4	IIB	GQ868585	Colombia	2005
DENV-4	IIB	HQ332176	Venezuela	2007
DENV-4	IIB	JF262782	Haiti	1994
DENV-4	IIB	KP188566	Brazil	2013
DENV-4	IIB	KT749994.1	Thailand	2011
DENV-4	IIB	MZ505611.1	Thailand	2015
DENV-4	III	AY618988	Thailand	1997
DENV-4	III	AY618989.1	Thailand_Bangkok	1997
DENV-4	III	KY586945.1	Thailand_Bangkok	1998
DENV-4	III	KY586946	Thailand	1998

DENV-4	I	AY618968.1	Thailand_Bangkok	1992
DENV-4	I	MW945505.1	Thailand	2011
DENV-4	I	MW945637.1	Thailand	2012
DENV-4	I	AY618990.1	Thailand_Bangkok	1991
DENV-4	I	AY618991	Thailand_Bangkok	1977
DENV-4	I	AY618992	Thailand_Bangkok	2001
Serotype	Genotype	Accession Number	Country	Year
DENV-4	I	AY947539.1	Philippines	1956
DENV-4	I	FJ196850	China	1990
DENV-4	I	JN638570	Cambodia	2008
DENV-4	I	JQ513345	Brazil	2011
DENV-4	I	KF041260	Pakistan	2009
DENV-4	I	KJ470764.1	Myanmar	2013
DENV-4	I	KP792537.2	Singapore	2011
DENV-4	I	KR011349	Philippines	1956
DENV-4	I	KR922405.1	Thailand	2011
DENV-4	I	KU509287	India	2009
DENV-4	I	KX845005	India	2015
DENV-4	I	KY234182.1	Thailand	2013
DENV-4	I	KY451945.1	Thailand_Chanthaburi	2013
DENV-4	I	KY851724.1	Thailand	2013
DENV-4	I	LC410197.1	Thailand	2016
DENV-4	I	LC410199.1	Thailand	2016
DENV-4	I	LC410201.1	Thailand	2016
DENV-4	I	LC410203.1	Thailand	2017
DENV-4	I	MG601754.1	China	2013
DENV-4	I	MG895367.1	Taiwan, Thailand	2015
DENV-4	I	MG895369.1	Taiwan, Thailand	2015
DENV-4	I	MG895372.1	Taiwan, Thailand	2015
DENV-4	I	MG895391.1	Taiwan, Thailand	2016
DENV-4	I	MH893694.1	Thailand	2016
DENV-4	I	MK780891.1	Southern Thailand	2016
DENV-4	I	MK780894.1	Southern Thailand	2016
DENV-4	I	MK780896.1	Southern Thailand	2016
DENV-4	I	MK780897.1	Southern Thailand	2016
DENV-4	I	MN955689.1	Thailand	2018
DENV-4	I	MN955691.1	Thailand	2018
DENV-4	I	MN955693.1	Thailand	2018
DENV-4	I	MT122862.1	Laos_Vientiane	2016
DENV-4	I	MT122879.1	Laos_Attapeu	2018
DENV-4	I	MT122896.1	Laos_Vientiane	2019
DENV-4	I	MW945624.1	Thailand	2014
DENV-4	I	MZ505612.1	Thailand	2015
DENV-4	I	MT524506.1	Northeastern Thailand	2016
DENV-4	I	MT524511.1	Northeastern Thailand	2017
DENV-4	I	MT524512.1	Northeastern Thailand	2016
DENV-4	I	MT524513.1	Northeastern Thailand	2016
DENV-4	I	MW793459.1	Thailand	2021
DENV-4	I	MW793460.1	Thailand	2021
DENV-4	I	MN955687.1	Thailand	2018
DENV-4	I	MN955688.1	Thailand	2018

DENV-4	I	MN955690.1	Thailand	2018
DENV-4	I	MN955692.1	Thailand	2018
DENV-4	I	MK780889.1	Southern Thailand	2015
DENV-4	I	MK780892.1	Southern Thailand	2016
DENV-4	I	MK780893.1	Southern Thailand	2016
DENV-4	I	MK780895.1	Southern Thailand	2016
DENV-4	I	MK780900.1	Southern Thailand	2015
Serotype	Genotype	Accession Number	Country	Year
DENV-4	I	MK780902.1	Southern Thailand	2015
DENV-4	I	KT825074.1	Thailand	2015
DENV-4	I	MT122877.1	Laos	2017
DENV-4	I	MT122869.1	Laos	2017
DENV-4	I	MT122865.1	Laos	2017
DENV-4	I	MT122873.1	Laos	2017
DENV-4	I	MT122881.1	Laos	2018
DENV-4	I	MT122878.1	Laos	2017
DENV-4	I	MT122857.1	Laos	2015
DENV-4	I	MT122894.1	Laos	2019
DENV-4	I	MT122895.1	Laos	2019
DENV-4	I	MT122884.1	Laos	2018
DENV-4	I	MT122893.1	Laos	2019
DENV-4	I	MT122886.1	Laos	2018
DENV-4	I	MT122875.1	Laos	2017
DENV-4	I	MT122863.1	Laos	2016
DENV-4	I	MT122858.1	Laos	2016
DENV-4	I	MT122872.1	Laos	2017
DENV-4	I	MK629501.1	Indonesia	2015
DENV-4	I	KY427081.1	Indonesia	2016
DENV-4	I	MF598872.1	China	2016
DENV-4	I	MF598870.1	China	2016
DENV-4	I	MF598869.1	China	2016
DENV-4	I	MG895395.1	Myanmar	2016
DENV-4	I	MH893696.1	China	2016
DENV-4	I	MG895333.1	Taiwan, Thailand	2013
DENV-4	I	MK614088.1	China	2019
DENV-4	I	MW295825.1	China	2017
DENV-4	I	KR051895.1	Myanmar	2013
DENV-4	I	MW945532.1	Thailand	2013
DENV-4	I	MG895351.1	Taiwan, Myanmar	2014
DENV-4	I	MH893695.1	Myanmar	2016

Table S4. Characteristics of DENV samples analyzed in the present study from Hospital for Tropical Diseases, Bangkok, Thailand, during 2018–2020.

ID	Collection Date	NS1 Ag	DENV IgM	DENV IgG	Serotype	Genotype	Accession No.
DV1I-PW-03-2018	November 28, 2018	NA	NA	NA	1	I	MZ618966
DV1I-PW-05-2018	December 12, 2018	NA	NA	NA	1	I	MZ618967
DV1I-PW-07-2019	January 11, 2019	NA	NA	NA	1	I	MZ618968
DV1I-PW-08-2019	January 16, 2019	NA	NA	NA	1	I	MZ618969
DV1I-PW-09-2019	January 15, 2019	NA	NA	NA	1	I	MZ618970
DV1I-TM18-18	May 25, 2018	Positive	NA	NA	1	I	MZ618971
DV1I-TM18-20	May 28, 2018	Positive	NA	NA	1	I	MZ618972
DV1I-TM18-57	August 29, 2018	Positive	NA	NA	1	I	MZ618973
DV1I-TM18-71	September 30, 2018	Positive	NA	NA	1	I	MZ618974
DV1I-TM18-75	October 10, 2018	Positive	NA	NA	1	I	MZ618975
DV1I-TM18-78	October 19, 2018	Positive	Negative	Negative	1	I	MZ618976
DV1I-TM18-79	October 24, 2018	Positive	NA	NA	1	I	MZ618977
DV1I-TM19-03	November 13, 2019	Positive	Positive	Positive	1	I	MZ618978
DV1I-TM19-15	November 19, 2019	Positive	NA	NA	1	I	MZ618979
DV1I-TM19-17	November 20, 2019	Positive	NA	NA	1	I	MZ618980
DV1I-TM19-24	November 22, 2019	Positive	NA	NA	1	I	MZ618981
DV1I-TM19-28	November 25, 2019	Positive	Negative	Negative	1	I	MZ618982
DV1I-TM19-36	November 27, 2019	Positive	NA	NA	1	I	MZ618983
DV1I-TM19-45	December 5, 2019	Positive	Positive	Positive	1	I	MZ618984
DV1I-TM19-50	December 10, 2019	Positive	Negative	Negative	1	I	MZ618985
DV1I-TM19-52	December 10, 2019	Positive	NA	NA	1	I	MZ618986
DV1I-TM19-54	December 11, 2019	Positive	NA	NA	1	I	MZ618987
DV1I-TM19-60	December 12, 2019	Positive	NA	NA	1	I	MZ618988
DV1I-TM19-69	December 22, 2019	Positive	Positive	Positive	1	I	MZ618989
DV1I-TM19-75	December 28, 2019	Positive	NA	NA	1	I	MZ618990
DV1I-TM19-79	December 30, 2019	Positive	NA	NA	1	I	MZ618991
DV1I-TM20-08	January 11, 2020	Positive	NA	NA	1	I	MZ618992
DV1I-TM20-12	January 12, 2020	Positive	NA	NA	1	I	MZ618993
DV1I-TM20-16	February 5, 2020	Positive	Negative	Negative	1	I	MZ618994
DV1I-TM20-20	February 16, 2020	Positive	NA	NA	1	I	MZ618995
DV1I-TM20-21	February 16, 2020	Positive	Negative	Negative	1	I	MZ618996
DV1I-TM20-22	June 11, 2020	Positive	NA	NA	1	I	MZ618997
DV1I-TM20-23	June 21, 2020	Positive	NA	NA	1	I	MZ618998
DV1I-TM20-24	June 27, 2020	Positive	NA	NA	1	I	MZ618999
DV1I-TM20-25	July 5, 2020	Positive	NA	NA	1	I	MZ619000
DV1I-TM20-30	July 21, 2020	Positive	NA	NA	1	I	MZ619001
DV1I-TM20-32	July 23, 2020	Positive	NA	NA	1	I	MZ619002
DV1I-TM20-44	August 17, 2020	Positive	NA	NA	1	I	MZ619003
DV1I-TM20-45	August 18, 2020	Positive	Positive	Negative	1	I	MZ619004
DV1I-TM20-47	August 19, 2020	Positive	NA	NA	1	I	MZ619005
DV1I-TM19-09-NGS	November 16, 2019	Positive	Negative	Negative	1	I	MZ619036
DV1I-TM19-12-NGS	November 19, 2019	Positive	NA	NA	1	I	MZ619037
DV1I-TM19-33-NGS	November 27, 2019	Positive	NA	NA	1	I	MZ619038
DV1I-TM19-40-NGS	November 29, 2019	Positive	NA	NA	1	I	MZ619039
DV1I-TM19-70-NGS	December 23, 2019	Positive	NA	NA	1	I	MZ619040
DV1I-TM19-74-NGS	December 28, 2019	Positive	Positive	Positive	1	I	MZ619041
DV2A-PW12	November 25, 2018	NA	NA	NA	2	Asian I	MZ636761
DV2A-PW14	December 1, 2018	NA	NA	NA	2	Asian I	MZ636762

DV2C-TM18-14	May 14, 2018	Positive	NA	NA	2	Cosmopolitan	MZ636763
**ID**	**Collection Date**	**NS1 Ag**	**DENV IgM**	**DENV IgG**	**Serotype**	**Genotype**	**Accession No.**
DV2C-TM18-15	May 21, 2018	Positive	Negative	Negative	2	Cosmopolitan	MZ636764
DV2C-TM18-16	May 22, 2018	Positive	NA	NA	2	Cosmopolitan	MZ636765
DV2C-TM18-33	July 5, 2018	Positive	NA	NA	2	Cosmopolitan	MZ636766
DV2C-TM18-36	July 13, 2018	Positive	Negative	Negative	2	Cosmopolitan	MZ636767
DV2C-TM18-47	August 13, 2018	Positive	Negative	Negative	2	Cosmopolitan	MZ636768
DV2A-TM18-66	September 17, 2018	Positive	NA	NA	2	Asian I	MZ636769
DV2C-TM18-86	November 16, 2018	Positive	NA	NA	2	Cosmopolitan	MZ636770
DV2C-TM19-11	November 19, 2019	Positive	NA	NA	2	Cosmopolitan	MZ636771
DV2C-TM19-16	November 19, 2019	Positive	Negative	Negative	2	Cosmopolitan	MZ636772
DV2C-TM19-19	November 20, 2019	Positive	Positive	Positive	2	Cosmopolitan	MZ636773
DV2C-TM19-23	November 21, 2019	Positive	NA	NA	2	Cosmopolitan	MZ636774
DV2C-TM19-29	November 25, 2019	Positive	NA	NA	2	Cosmopolitan	MZ636775
DV2C-TM19-31	November 26, 2019	Positive	NA	NA	2	Cosmopolitan	MZ636776
DV2C-TM19-32	November 26, 2019	Positive	NA	NA	2	Cosmopolitan	MZ636777
DV2C-TM19-35	November 27, 2019	Positive	Positive	Positive	2	Cosmopolitan	MZ636778
DV2C-TM19-42	December 1, 2019	Positive	NA	NA	2	Cosmopolitan	MZ636779
DV2A-TM19-43	December 3, 2019	Positive	NA	NA	2	Asian I	MZ636780
DV2C-TM19-46	December 5, 2019	Positive	NA	NA	2	Cosmopolitan	MZ636781
DV2A-TM19-63	December 16, 2019	Positive	NA	NA	2	Asian I	MZ636782
DV2C-TM19-73	December 28, 2019	Positive	NA	NA	2	Cosmopolitan	MZ636783
DV2C-TM19-81	December 31, 2019	Positive	NA	NA	2	Cosmopolitan	MZ636784
DV2C-TM20-11	January 12, 2020	Positive	NA	NA	2	Cosmopolitan	MZ636785
DV2A-TM20-33	July 27, 2020	Positive	NA	NA	2	Asian I	MZ636786
DV2C-TM20-36	August 3, 2020	Positive	NA	NA	2	Cosmopolitan	MZ636787
DV2C-TM20-37	August 8, 2020	Positive	Negative	Negative	2	Cosmopolitan	MZ636788
DV2A-TM20-43	August 16, 2020	Positive	Negative	Negative	2	Asian I	MZ636789
DV2C-TM20-55	August 29, 2020	Positive	Negative	Negative	2	Cosmopolitan	MZ636790
DV2C-TM20-56	August 31, 2020	Positive	Negative	Negative	2	Cosmopolitan	MZ636791
DV2C-TM20-65	September 22, 2020	Positive	NA	NA	2	Cosmopolitan	MZ636792
DV2C-TM20-74	October 3, 2020	Positive	NA	NA	2	Cosmopolitan	MZ636793
DV2A-TM20-83	October 9, 2020	Positive	NA	NA	2	Asian I	MZ636794
DV2A-TM20-89	October 20, 2020	Positive	NA	NA	2	Asian I	MZ636795
DV2C-TM20-93	October 26, 2020	Positive	Negative	Positive	2	Cosmopolitan	MZ636796
DV2A-TM20-94	October 30, 2020	Positive	NA	NA	2	Asian I	MZ636797
DV2A-TM20-96	November 3, 2020	Positive	NA	NA	2	Asian I	MZ636798
DV2A-TM20-101	November 13, 2020	Positive	NA	NA	2	Asian I	MZ636799
DV2A-TM19-13-NGS	November 19, 2019	Positive	NA	NA	2	Asian I	MZ636801
DV2C-TM19-26-NGS	November 24, 2019	Positive	NA	NA	2	Cosmopolitan	MZ636802
DV2C-TM19-37-NGS	November 28, 2019	Positive	NA	NA	2	Cosmopolitan	MZ636803
DV2C-TM19-41-NGS	November 30, 2019	Positive	NA	NA	2	Cosmopolitan	MZ636804
DV2C-TM19-80-NGS	December 30, 2019	Positive	NA	NA	2	Cosmopolitan	MZ636805
DV3I-PW23	October 10, 2018	NA	NA	NA	3	I	MZ636813
DV3III-TM18-12	May 8, 2018	Positive	Negative	Negative	3	III	MZ636814
DV3I-TM18-22	June 4, 2018	Positive	Negative	Negative	3	I	MZ636815
DV3I-TM18-34	July 7, 2018	Positive	Negative	Negative	3	I	MZ636816
DV3I-TM20-02	January 2, 2020	Positive	NA	NA	3	I	MZ636817
DV3III-TM20-07	January 10, 2020	Positive	NA	NA	3	III	MZ636818
DV3I-TM20-09	January 11, 2020	Positive	NA	NA	3	I	MZ636819
DV3I-TM20-10	January 11, 2020	Positive	NA	NA	3	I	MZ636820

ID	Collection Date	NS1 Ag	DENV IgM	DENV IgG	Serotype	Genotype	Accession No.
DV4I-PW-11	December 1, 2018	NA	NA	NA	4	I	MZ636822
DV4I-PW-34	November 1, 2018	NA	NA	NA	4	I	MZ636823
DV4I-PW-37	November 1, 2018	NA	NA	NA	4	I	MZ636824
DV4I-PW-44	December 1, 2018	NA	NA	NA	4	I	MZ636825
DV4I-TM18-7	March 27, 2018	Positive	NA	NA	4	I	MZ636826
DV4I-TM19-76	December 29, 2019	Positive	NA	NA	4	I	MZ636827
DV4I-TM20-5	January 9, 2020	Positive	NA	NA	4	I	MZ636828
DV4I-TM20-53	August 29, 2020	Positive	Positive	Positive	4	I	MZ636829
DV4I-TM20-59	September 11, 2020	Positive	NA	NA	4	I	MZ636830
DV4I-TM20-64	September 18, 2020	Positive	Negative	Positive	4	I	MZ636831
DV4I-TM20-76	October 3, 2020	Positive	NA	NA	4	I	MZ636832
DV4I-TM20-90	October 21, 2020	Positive	Negative	Negative	4	I	MZ636833
DV4I-TM20-98	November 9, 2020	Positive	Negative	Positive	4	I	MZ636834

NA, data not available.
